# Supplementary material for: Eating Fast Has a Significant Impact on Glycemic Excursion in Healthy Women: Randomized Controlled Cross-Over Trial
Source: Nutrients. 2020 Sep 10;12(9):2767. doi: 10.3390/nu12092767 (PMC7551722; doi:10.3390/nu12092767)
Supplement: Supplementary file 1 [file nutrients-12-02767-s001.pdf]

Table S1. The composition and macronutrient content of the menu of the test meals.

Meals			Weight (g)	Energy (kcal)	Protein (g)	Fat (g)	Carbohydrate (g)	Fiber (g)	Salt (g)
Breakfast	White bread		90	238	8.4	4.0	42.0	2.1	1.2
	Broccoli		60	16	2.1	0.2	2.6	2.2	0.0
	Tomato		150	29	1.1	0.2	7.1	1.5	0.0
	Milk		200	134	6.6	7.6	9.6	0.0	0.2
	Strawberry jam (sugar-free)		13	20			8.8		
		Total of breakfast		437	18.2	12.0	70.1	5.8	1.4
Lunch	Boiled white rice		200	336	5.0	0.6	74.2	0.6	0.0
	Tomato		150	29	1.1	0.2	7.1	1.5	0.0
	Boiled vegetables	Spinach	80	20	2.1	0.4	3.2	2.9	0.0
		Soy sauce	6	4	0.5	0.0	0.6	0.0	0.9
		Bonito soup	5	0	0.0	0.0	0.0	0.0	0.0
	Frozen meal box of fried fish with vegetable	Fish, oil, onion, carrot, radish paprika	250	235	16.4	10.3	18.9	3.1	2.0
		Total of lunch		624	25.1	11.5	104.0	8.1	2.9
Dinner	Boiled white rice		200	336	5.0	0.6	74.2	0.6	0.0
	Tomato		150	29	1.1	0.2	7.1	1.5	0.0
	Boiled vegetables	Spinach	80	12	1.3	0.1	2.4	1.9	0.0
		Fried tofu	15	27	1.9	2.1	0.0	0.1	0.0
		Bonito soup	15	0	0.0	0.0	0.0	0.0	0.0
		Soy sauce	6	4	0.5	0.0	0.6	0.0	0.9
	Frozen meal box of gluten steak with vegetable	Gluten-meat steak, carrot, radish, onion	250	281	13.8	14.4	23.3	3.7	1.6
		Total of dinner		689	23.6	17.4	107.6	7.8	2.5
		Total		1750	66.9	40.9	281.7	21.7	6.8
